# Modeling of Al and Ga Droplet Nucleation during Droplet Epitaxy or Droplet Etching

**DOI:** 10.3390/nano11020468

**Published:** 2021-02-12

**Authors:** Christian Heyn, Stefan Feddersen

**Affiliations:** Center for Hybrid Nanostructures (CHyN), University of Hamburg, Luruper Chaussee 149, 22761 Hamburg, Germany; stefan.feddersen@studium.uni-hamburg.de

**Keywords:** droplet density, droplet epitaxy, droplet etching, nucleation, scaling, rate model, Monte Carlo simulation

## Abstract

The temperature dependent density of Al and Ga droplets deposited on AlGaAs with molecular beam epitaxy is studied theoretically. Such droplets are important for applications in quantum information technology and can be functionalized e.g., by droplet epitaxy or droplet etching for the self-assembled generation of quantum emitters. After an estimation based on a scaling analysis, the droplet densities are simulated using first a mean-field rate model and second a kinetic Monte Carlo (KMC) simulation basing on an atomistic representation of the mobile adatoms. The modeling of droplet nucleation with a very high surface activity of the adatoms and ultra-low droplet densities down to 5 × 10${}^{6}$ cm${}^{-2}$ is highly demanding in particular for the KMC simulation. Both models consider two material related model parameters, the energy barrier $E_{S}$ for surface diffusion of free adatoms and the energy barrier $E_{E}$ for escape of atoms from droplets. The rate model quantitatively reproduces the droplet densities with $E_{S}$ = 0.19 eV, $E_{E}$ = 1.71 eV for Al droplets and $E_{S}$ = 0.115 eV for Ga droplets. For Ga, the values of $E_{E}$ are temperature dependent indicating the relevance of additional processes. Interestingly, the critical nucleus size depends on deposition time, which conflicts with the assumptions of the scaling model. Using a multiscale KMC algorithm to substantially shorten the computation times, Al droplets up to 460 °C on a 7500 × 7500 simulation field and Ga droplets up to 550 °C are simulated. The results show a very good agreement with the experiments using $E_{S}$ = 0.19 eV, $E_{E}$ = 1.44 eV for Al, and $E_{S}$ = 0.115 eV, $E_{E}$ = 1.24 eV (T≤ 300 °C) or $E_{E}$ = 1.24 + 0.06 (*T*[°C] − 300)/100 eV (T>300 °C) for Ga. The deviating $E_{E}$ is attributed to a re-nucleation effect that is not considered in the mean-field assumption of the rate model.

## 1. Introduction

Semiconductor quantum dots (QDs) are established as quantum emitters and represent essential building blocks for quantum information technology [1,2,3]. For their fabrication, the use of self-assembly techniques is a powerful approach providing versatile semiconductor nanostructures by molecular beam epitaxy (MBE) [4]. Two major paths are pursuit relying on different mechanisms for the self-assembly. In the Stranski-Krastanov growth, epitaxial layers of different lattice constant are deposited and the driving force for self-assembled formation of e.g., InAs/GaAs QDs or Ge/Si QDs is the minimization of the strain energy [5,6,7]. As a drawback of this technique, the resulting QDs are substantially strained. More flexible regarding the choice of the materials are droplet-based techniques [8] such as the droplet epitaxy [9,10,11,12,13] or droplet etching [14,15,16,17,18]. Here, the driving force for self-assembly is the minimization of the solid and liquid surface and interface energies. For droplet epitaxy, often Ga droplets are deposited on AlGaAs surfaces and subsequently recrystallized to form GaAs QDs [19,20] or quantum rings [21]. For droplet etching, usually Al droplets are deposited on AlGaAs, transformed into low-density nanoholes during post-growth annealing, and subsequently filled with GaAs to form GaAs QDs [22,23] or quantum dot molecules [24].

For both methods, droplet epitaxy and droplet etching, the desired feature density is equal to the density of the initial droplets. Thus, the precise control of the droplet density is essential for the creation of quantum emitters with well-defined properties. For instance, low density QDs [25] are relevant as single-photon sources for quantum information technology and high density dots [26] for device applications such as lasers or solar cells. The central process parameter controlling the droplet density is the substrate temperature *T*. In an earlier study [12], we have discussed the *T*-dependent density of Ga droplets, but only for low temperatures up to 300 °C and using only basic approaches for modeling. Here, we study also Al droplets and significantly expand the temperature range to consider technologically relevant processes. Furthermore, we compare three different approaches for the modeling of the experimental data. In this sense, the present manuscript follows two goals: first, the better understanding of the experimental droplet densities and the determination of relevant material parameters and, second, the evaluation of different concepts for droplet modeling.

Popular approaches for modeling of nucleation on surfaces during crystal growth are coupled mean-field rate equations [12,27,28,29] and kinetic Monte Carlo (KMC) simulations [30,31,32,33]. Rate equations describe the time-dependent average density of objects on the surface such as mobile adatoms (monomers) and clusters of various size. In principle, every cluster size requires the calculation of an individual equation which can be very time-consuming for system containing large clusters. To overcome this issue, a critical cluster size *i* is introduced which represents the smallest stable cluster size [27,28,29]. Clusters with size below *i* are unstable and the monomer escape rate is larger than the rate of attachment. Now, all stable clusters can be averaged and do not require individual equations.

As an advantage in comparison to a mean-file rate model, a kinetic Monte Carlo simulation is based on an atomistic representation of the surface adatoms and allows a modeling also of the droplet size distribution. On the other side, the computation time of a KMC simulation substantially increases for large simulation fields (required for the present low droplet densities) and for a high rate of monomer diffusion processes. This limits the maximal process temperature accessible by this method. Starting with a conventional KMC approach, we have simulated the nucleation of Al droplets up to a temperature *T* of 300 °C. By using a multiscale KMC algorithm [32], we have reduced the computation time by a factor of 30,000 in comparison to a conventional KMC approach and achieved very good agreement with experimental Al droplet densities up to a temperature of 460 °C on a 7500 × 7500 simulation field and with Ga droplets up to 550 °C.

## 2. Experimental Droplet Density

**Figure 1:**
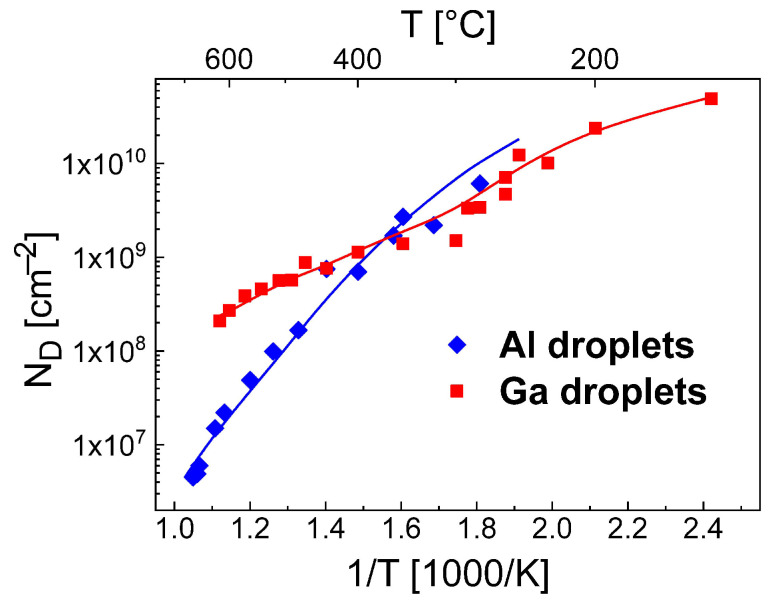
Density $N_{D}$ of Al and Ga droplets as function of temperature *T*. The experimental data (symbols) are taken from AFM images [34,35]. The lines are calculated using the rate model with $E_{S}$ = 0.19 eV, $E_{E}$ = 1.71 eV for Al droplets and $E_{S}$ = 0.115 eV, $E_{E}$ = *T*-dependent (see text) for Ga droplets.

The sample fabrication using solid-source MBE on (001) GaAs wafers is described previously [34,35]. In brief, after deposition of an atomically flat AlGaAs layer, the As flux is reduced and the droplet material is deposited. For Ga, the material flux to the surface is *F* = 0.8 mL/s, the deposition time = 4 s, and the resulting coverage of droplet material on the substrate surface is Ft = 3.2 mL. For Al, the parameters are *F* = 0.4 mL/s, deposition time = 2.5 s, and coverage Ft = 1.0 mL. The droplets are formed in Volmer-Weber growth mode [36] driven by the minimization of the liquid and solid surface energies and of the liquid-solid interface energy. For higher process temperatures [35], the deposited droplets drill nanoholes into the substrate, which is called droplet etching [14,15,16,17,18]. Relevant processes are here the diffusion of As from the crystalline substrate into the droplet material and the removal of the droplet material by spreading over the substrate surface [37]. Importantly, since every droplet forms a nanohole [17], the initial droplet density can be determined from nanohole data. After growth, the surfaces are characterized using atomic force microscopy (AFM) and the droplet densities $N_{D}$ are determined. Figure 1 shows values of $N_{D}$ after deposition of Ga and Al as function of the temperature *T* during droplet deposition. The data are taken from previous publications [34,35].

## 3. Computation

The computations are performed on personal computers. We have started the simulations with the rate equations model using the interpreted programming language Python3. To reduce the computation time we have tested Julia (about two time faster than Python) and finally switched to the compiled languages Delphi (about 10 time faster than Python) and C++ (about 1.5 times faster than Delphi). The final computations of both the rate equations model as well as the kinetic Monte Carlo simulation are done parallel using two codes one in Delphi and one in C++. Typical computation times of the rate model range from a few minutes up to a few hours and depend on the simulated process temperature. The computation time is determined by the accuracy of the time interval for the numerical integration, where process conditions with a smaller monomer density require a smaller time interval. On the other side, the computation times of the KMC simulations are much longer and depend crucially on the simulated process temperature, since at a higher *T* the rate of surface activity increases and the reduced droplet densities require larger simulation fields. A conventional KMC simulation for a process temperature of *T* = 300 °C using a 1400 × 1400 simulation field takes about one week, which we consider as a limit for a reasonable parameterization. A multiscale KMC approach (see below) computes the same process substantially faster in about 20 s. Here, the limit for Al droplets are simulations for *T* = 460 °C using a 7500 × 7500 simulation field and for Ga droplets *T* = 550 °C, which require more than one week.

## 4. Scaling Analysis

We start the analysis of the experimental droplet density using classical nucleation theory [27,28,29], which predicts the density of stable three-dimensional clusters by a scaling law

(1) $$N_{D}\propto F^{p}\exp\left[E/\left(k_{B}T\right)\right]$$

For complete condensation of three-dimensional clusters the scaling energy becomes $E=p\left(E_{S}+E_{i}/i\right)$, with the energy barrier $E_{S}$ for surface diffusion, $p=i/\left(i+2.5\right)$, the critical cluster size *i*, and the energy $E_{i}$ of a critical cluster.

A scaling analysis of the experimental droplet densities in Figure 1 yields a fitted slope of *E* = 0.74 eV for Al (*T* = 280...570 °C) and *E* = 0.35 eV for Ga droplets. The reduction of $N_{D}$ at T> 570 °C for Al droplets was earlier [12] related to droplet coarsening by Ostwald ripening [29,38].

Earlier flux dependent data [12] indicate *p* = 0.5 and thus *i* = 2.5 for a low *T* = 200 °C and Ga droplets. We will show below that this value of *i* is not valid for the higher temperatures discussed here. For a comparison with the rate model described below, an energy barrier $E_{E}$ for escape of atoms from a cluster is introduced with $E_{E}=2E_{i}/i$ [12]. Now, the scaling energy becomes $E=p\left(E_{S}+E_{E}/2\right)$.

## 5. Mean-Field Rate Equations

Mean-field rate equations represent a common approach to model nucleation processes during crystal growth [12,27,28,29]. The present model considers the average densities of three types of objects on the growing surface: (1) Mobile adatoms (monomers) with density $N_{1}$. (2) Small droplet-like immobile clusters of density $N_{s}$ that are composed of *s* atoms and potentially unstable, i.e., below the critical cluster size *i*. (3) Droplets with density $N_{D}$ and an average volume of *s* atoms that are above the critical cluster size and, thus, stable. Droplets and clusters are approximated by a hemispherical shape.

Furthermore, three classes of processes are considered: (1) Arrival of the impinging material with a flux *F* on the surface and formation of monomers. (2) Attachment of mobile monomers to either other monomers, to clusters with size *s*, or to droplets. The corresponding rates are $R_{A,1}$, $R_{A,s}$, and $R_{A,D}$, respectively. In this scheme, collisions between mobile adatoms represent nucleation events. (3) Escape of monomers from clusters or droplets with the respective rates $R_{E,s}$ and $R_{E,D}$.

The driving force for monomer mobility and their attachment to other objects is the surface diffusion where the hopping frequency is given by the diffusion coefficient $D=\nu \exp\left[-E_{S}/\left(k_{B}T\right)\right]$, with $\nu =2k_{B}T/h$ is a vibrational frequency [39], $k_{B}$ Boltzmann’s constant, and *h* Planck’s constant. We have to consider also the capture numbers [27,28,40] $\sigma_{s}$, $\sigma_{D}=2\pi \left(r/\lambda \right)k_{1}\left(r/\lambda \right)/k_{0}\left(r/\lambda \right)$, which reflects the depletion of the monomer density around monomers, clusters, or droplets, where $k_{0}$, $k_{1}$ are modified Bessel functions, *r* is the radius, and $\lambda^{-2}=\left(F/D\right)+2\sigma_{1}N_{1}+\sigma_{D}N_{D}$ is the surface diffusion length [40]. We assume that clusters and droplets are hemispherically shaped with a radius of $r=\sqrt[{3}]{3s/(2\pi )}$. This allows to calculate the monomer attachment rates to other monomers (*s* = 1) and clusters with size *s* as $R_{A,s}=N_{1}\sigma_{s}N_{s}D$, and to droplets as $R_{A,D}=N_{1}\sigma_{D}N_{D}D$.

Escape of atoms from clusters with size *s* is considered with rate $R_{E,s}=2\pi r\zeta N_{s}D_{E}$ and from droplets with rate $R_{D}=2\pi r\zeta N_{D}D_{E}$, where $D_{E}=\nu \exp\left[-E_{E}/\left(k_{B}T\right)\right]$, $\zeta =\exp(r_{c}/r)$ describes the enhancement of the vapor pressure for small droplets due to the Gibbs-Thomson effect, $r_{c}=2\gamma V_{mol}/\left(N_{A}k_{B}T\right)$, γ is the surface tension (0.89 N/m for Al and 0.67 N/m for Ga), $V_{mol}$ the molar volume (10.0 × 10${}^{-6}$ m${}^{3}$/mol for Al and 11.8 × 10${}^{-6}$ m${}^{3}$/mol for Ga), and $N_{A}$ the Avogadro constant.

In the following a mean-field model basing on a set of coupled rate-equations is introduced which describes the droplet nucleation and allows to calculate the time dependence of the average droplet density. Since the critical cluster size *i* depends on the model parameters and is not know initially, a maximum possible unstable cluster size j>i is introduced. The first equation describes the monomer density, which is balanced by the flux of impinging adatoms, monomer attachment to other monomers, clusters, and droplets, as well as by escape from clusters and droplets:

(2) $$dN_{1}\left(t\right)/t=F-2R_{A,1}-\sum_{s=2}^{j}R_{A,s}-R_{A,D}+2R_{E,2}+\sum_{s=3}^{j}R_{E,s}+R_{E,D}$$

The density of clusters with size of *s* = 2...j−1 atoms evolves as:

(3) $$dN_{s}\left(t\right)/dt=R_{A,s-1}-R_{A,s}-R_{E,s}+R_{E,s+1}$$

and that of clusters with s=j as:

(4) $$dN_{j}\left(t\right)/dt=R_{A,j-1}-R_{A,j}-R_{E,j}$$

Finally, the droplet density follows:

(5) $$dN_{D}\left(t\right)/dt=R_{A,j}$$

For the model calculations, the *j* + 1 individual rate equations (Equations (2)–(5)) are solved numerically (see Section 3).

**Figure 2:**
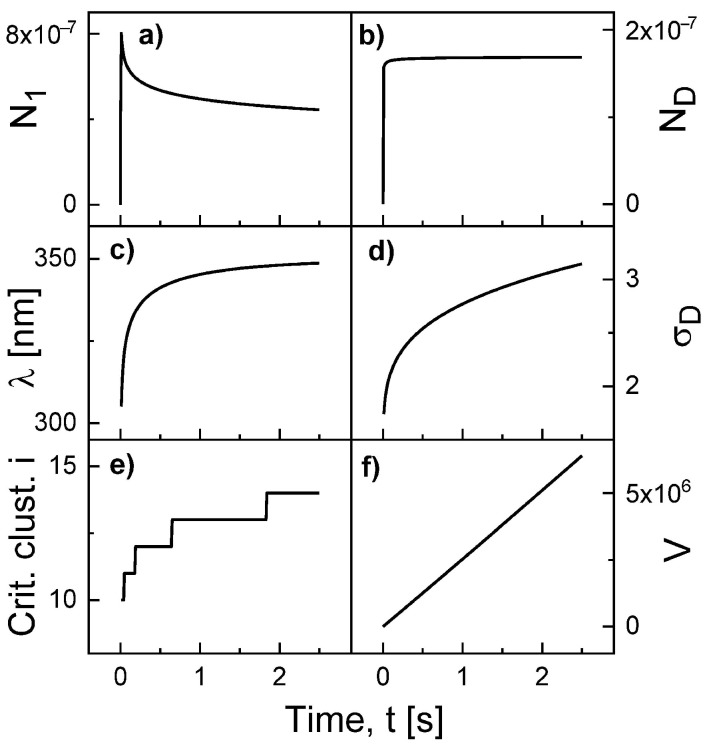
Simulated time evolution of various quantities during Al droplet deposition at *F* = 0.4 mL/s, deposition time 2.5 s, *T* = 500 °C, $E_{S}$ = 0.19 eV, and $E_{E}$ = 1.71 eV. (**a**) Monomer density $N_{1}$, (**b**) droplet density $N_{D}$, (**c**) surface diffusion length λ, (**d**) droplet capture number $\sigma_{D}$, (**e**) critical cluster size *i*, and (**f**) droplet volume *V* in atoms.

Figure 2 show an example for the simulated time evolution of various quantities during Al droplet deposition at *F* = 0.4 mL/s, deposition time 2.5 s, *T* = 500 °C, $E_{S}$ = 0.19 eV, and $E_{E}$ = 1.71 eV. The monomer density $N_{1}$ initially increases, followed by a decrease due to nucleation and attachment events. The droplet density $N_{D}$ saturates very fast. We note that this is not the case at low temperatures. Both the surface diffusion length λ = 300...350 nm and the capture number $\sigma_{D}$ = 1.4...3.1 increase slightly during deposition. As an interesting result, the critical cluster size *i* increases from 10 to 14. The critical cluster is the smallest cluster size *s* which fulfills the condition $R_{A,s}\geq R_{E,s}$. In the numerical calculations, *j* must be always larger then *i*. In addition, finally the droplet volume which increases nearly linear with time according to $V=s≃Ft/N_{D}$.

**Figure 3:**
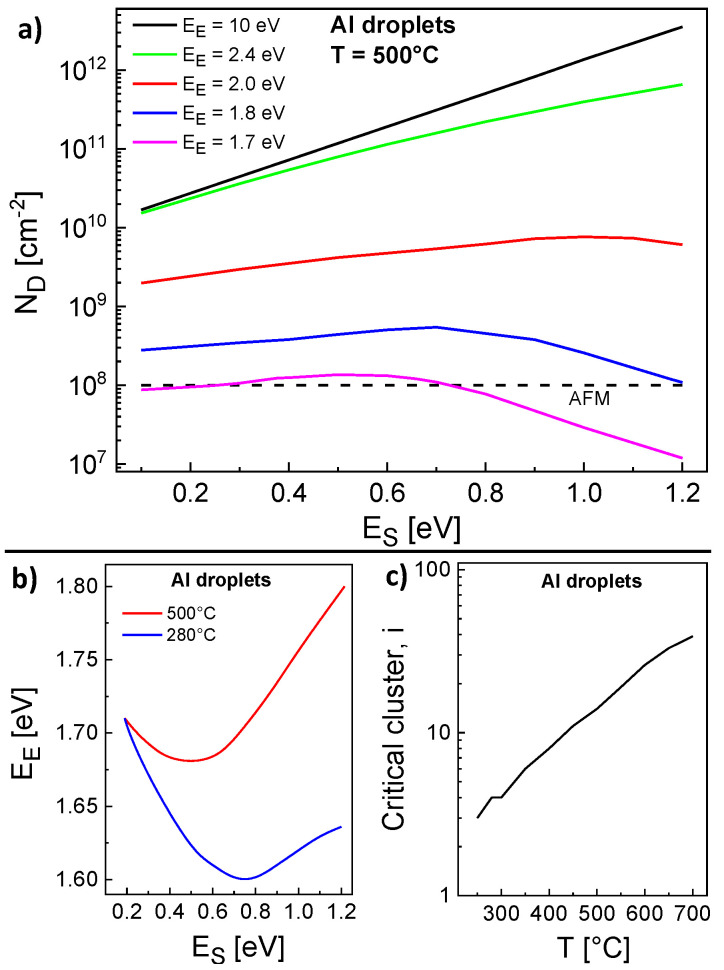
Parameterization of the rate model for Al droplets. (**a**) Calculated droplet densities $N_{D}$ for varied $E_{S}$ and $E_{E}$ at *T* = 500 °C. The dashed line indicates the experimental $N_{D}$ taken from AFM data. Agreement between experiment and model results is obtained for a range of pairs $E_{S}$, $E_{E}$. (**b**) Pairs $E_{S}$, $E_{E}$ with agreement to the experimental $N_{D}$ for *T* = 500 °C and *T* = 280 °C. For $E_{S}$ = 0.19 eV, $E_{E}$ = 1.71 eV agreement is achieved for both temperatures. (**c**) Critical cluster size *i* at the end of the deposition time as function of *T*.

The rate model has two free model parameters, the energy barrier $E_{S}$ for surface diffusion of monomers and the energy barrier $E_{E}$ for escape of monomers from clusters or droplets. We start with the parameterization of the model for the Al droplet density using the experimental flux *F* = 0.4 mL/s and a deposition time of 2.5 s, which yields a droplet material coverage Ft = 1.0 mL. In a first step, we have calculated $N_{D}$ for varied $E_{S}$ and $E_{E}$ at a constant *T* = 500 °C. Results are shown in Figure 3a and demonstrate that $N_{D}$ depends on $E_{S}$ and even stronger on $E_{E}$. Interestingly, the expected decrease of $N_{D}$ with decreasing $E_{S}$ is observed only for very large $E_{E}$ = 10 eV, i.e., irreversible aggregation. Here, the mobile adatoms perform a faster migration at a lower $E_{S}$ and attach preferred to existing islands instead of nucleating new ones. In contrast to that, a lower $E_{N}\leq$ 2.0 eV yields two regimes separated by a maximum of $N_{D}$. We assume that $E_{E}$ influences $N_{D}$ by two competing effects. First, the nucleation rate will be reduced due to the escape of atoms from clusters with size below *i*. Second, the monomer density is increased by escape which yields an enhanced nucleation rate. Therefore, the regime at lower $E_{S}$ is diffusion controlled and $N_{D}$ decreases with decreasing $E_{S}$, whereas the regime at higher $E_{S}$ is escape controlled and $N_{D}$ decreases with increasing $E_{S}$.

A comparison with droplet densities obtained from AFM measurements establish that a range of pairs $E_{S}$, $E_{E}$ provides agreement between model calculations and experiments (Figure 3a). A summary of the possible pairs is plotted in Figure 3b. The same procedure for *T* = 280 °C gives a second range of pairs $E_{S}$ and $E_{E}$ (Figure 3b). As a key point, there is only one pair $E_{S}$ = 0.19 eV, $E_{E}$ = 1.71 eV which yields agreement for both temperatures and represents an unambiguous determination of both energy values. Figure 1 demonstrates the very good reproduction of the experimental droplet densities by the rate model using these parameters.

As a further interesting point, the rate model reproduces also the experimental reduction of $N_{D}$ at T> 570 °C. This reduction was earlier [12] attributed to droplet coarsening by Ostwald ripening [29,38]. Since the present model does not consider droplet coarsening, the relevance of Ostwald ripening for droplet formation is now questionable.

In addition, Figure 3c shows that the temperature dependence of the critical cluster size *i* = 3...39 at the end of deposition follows approximately i∝exp(T). Since a time and *T*-dependent *i* is not considered in the scaling model (Equation (1)), a scaling analysis of the droplet densities establishes as an only rough approximation. Nevertheless, from $E=p\left(E_{S}+E_{E}/2\right)$ or $p=E/\left(E_{S}+E_{E}/2\right)$, an effective critical cluster size i= 6 can be estimated.

**Figure 4:**
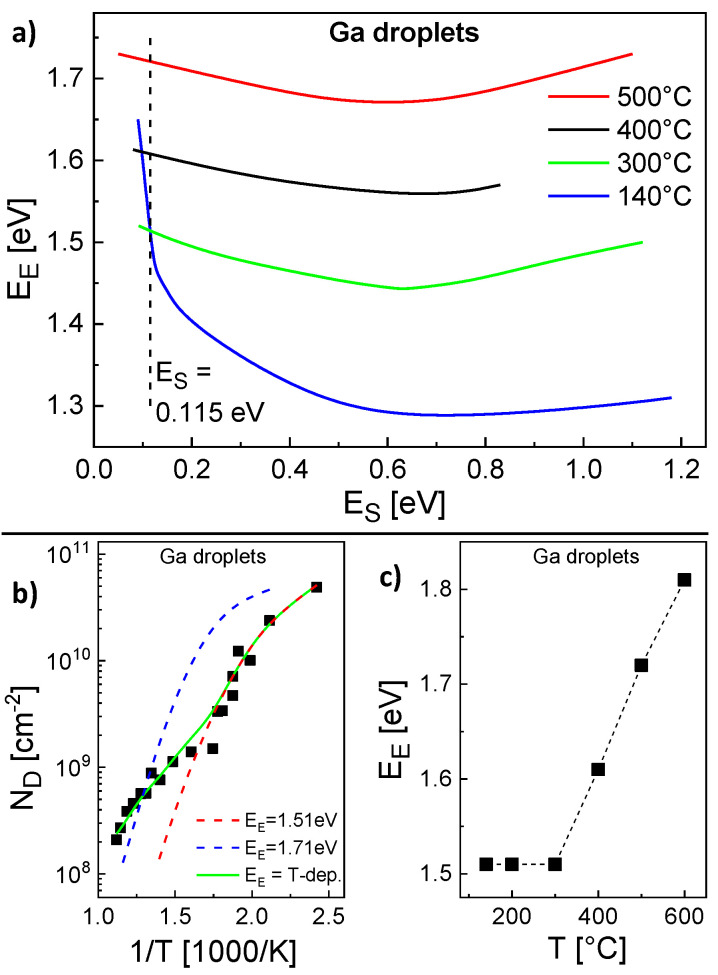
Parameterization of the rate model for Ga droplets. (**a**) Pairs $E_{S}$, $E_{E}$ with agreement to the experimental $N_{D}$ for several temperatures as indicated. (**b**) Experimental Ga droplet density (symbols) together with model results calculated using different parameters. $E_{S}$ is always 0.115 eV and $E_{E}$ is indicated. (**c**) *T*-dependent values of $E_{E}$ for agreement with the experimental $N_{D}$ at a constant $E_{S}$ = 0.115 eV.

The parameterization of the model for the Ga droplet density is more complex. Here, the experimental conditions are *F* = 0.8 mL/s and a deposition time = 4 s. Again, pairs $E_{S}$, $E_{E}$ with agreement between calculated and experiments $N_{D}$ are determined (Figure 4a). However, in contrast to the Al droplets above, for the Ga droplet density there is no pair which provides agreement for the whole temperature range. Only the low temperature regime from 140...300 °C is reproduced by a common pair $E_{S}$ = 0.115 eV, $E_{E}$ = 1.51 eV and calculations using these parameters agree well with the AFM data in this regime (Figure 4b). The behavior at higher temperatures cannot be reproduced using these energies (Figure 4b). We assume that above 300 °C either $E_{S}$ or $E_{E}$ depends on *T*. Since a variation of $E_{S}$ has an only small effect, probably $E_{E}$ is the relevant *T*-dependent parameter. Assuming a constant $E_{S}$ = 0.115 eV, Figure 4c shows values of $E_{E}$ which provide agreement with the experimental $N_{D}$ at varied *T*. Figure 4c clearly establishes that above 300 °C the experimental $N_{D}$ cannot be reproduced assuming a constant $E_{E}$. This is also confirmed by the example in Figure 4b which agrees only at *T* = 500 °C using $E_{S}$ = 0.115 eV and a constant $E_{E}$ = 1.71 eV. As a consequence, for a reproduction of the whole temperature range, the escape energy must be *T*-dependent with $E_{E}$ = 1.51 eV for T≤ 300 °C and $E_{E}$ = 1.51 + 0.1 (*T*[°C] − 300)/100 eV for T> 300 °C. This choice for $E_{E}$ allows a very good reproduction of the Ga droplet density (Figure 1 and Figure 4b).

An analysis of the scaling behavior makes sense only for T≤ 300 °C where $E_{E}$ is constant. Here, $p=E/\left(E_{S}+E_{E}/2\right)$ yields an effective critical cluster size of about i= 2.

## 6. Kinetic Monte Carlo Simulations

The kinetic Monte Carlo (KMC) simulation model considers in an atomistic picture the activity of mobile atoms (monomers) on the growing surface and their nucleation to droplets (size s≥ 2 atoms). The model assumes that only monomers are mobile. The surface is represented as a square simulation field with $m_{n}=m_{x}\times m_{y}$ elements and cyclic boundary conditions. The positions of the respective monomers and droplets as well a the individual droplet sizes define the status of the growth process.

We start with a conventional KMC approach (called MC1), where individual atoms can arrive on the surface with flux *F*, hop to a nearest-neighbor surface site with rate $D=\nu \exp\left[-E_{S}/\left(k_{B}T\right)\right]$, or escape from a hemispherically shaped droplet composed of *s* atoms with rate $R_{E,s}=2\pi r\zeta \nu \exp\left[-E_{E}/\left(k_{B}T\right)\right]$, *r* is the droplet radius, and ζ the Gibbs-Thomson enhancement as is already defined above for the rate model. In the MC1 model, the various rates sum up to a total activity rate:

(6) $$R_{tot}=m_{n}F+n_{1}D+\sum_{s}n_{s}R_{E,s}$$

with the monomer number $n_{1}$ and the numbers $n_{s}$ of droplets with size *s*. This allows the random selection of the next process as well as the calculation of the time interval up to the next event $dt=1/R_{tot}$.

**Figure 5:**
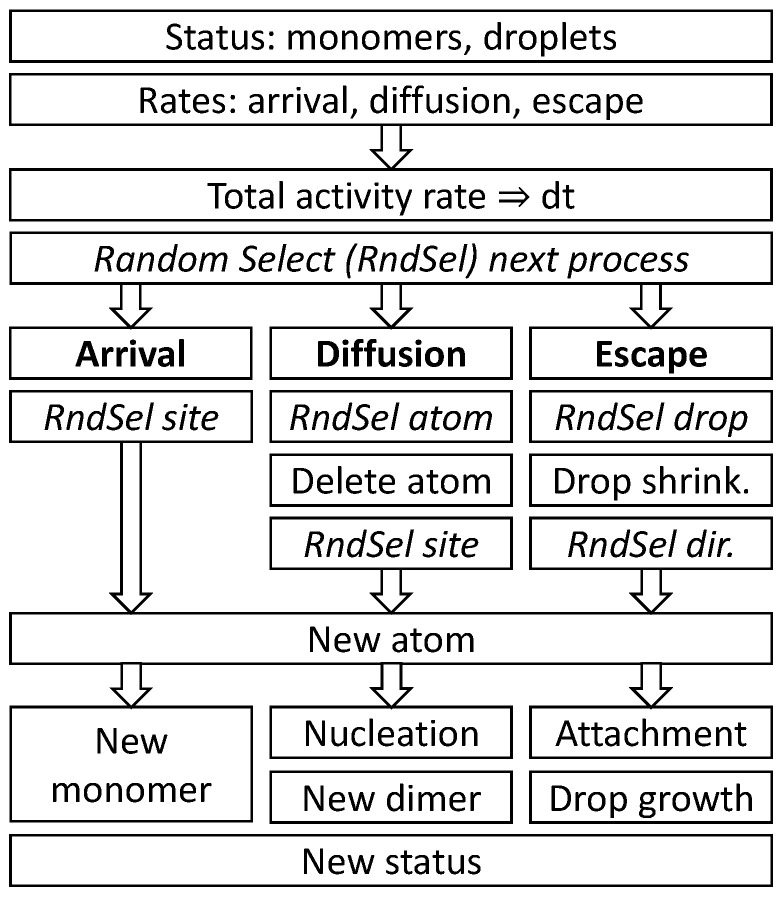
Scheme of a single loop of the kinetic Monte Carlo simulation model.

The arrival of an atom on the surface can result either in the formation of a new monomer, a nucleation event by a direct hit to another monomer, or in droplet growth by a direct hit. Monomer diffusion can cause a site-change, a nucleation event, or the attachment to a droplet. An escape from a droplet yields a new monomer at a random angle and distance *r* + 2 from the droplet center. A scheme of a single loop of the Monte Carlo model MC1 is shown in Figure 5.

For a reasonable statistics we consider a minimum of 20 droplets per simulation field. This requires for Al droplets, e.g., a 1400 × 1400 field for *T* = 300 °C, 4000 × 4000 for *T* = 400 °C, and 10,000 × 10,000 for *T* = 500 °C. Simulation runs for Al droplets at *T* = 300 °C are performed on a 1400 × 1400 grid using the energy values $E_{S}$ = 0.19 eV and $E_{E}$ = 1.71 eV as determined by the rate model above. The simulated droplet density of 2.3 × 10${}^{10}$ cm${}^{-2}$± 7% is significantly larger compared to the results of the rate model. This disagreement demonstrates that the determined energy values are model-dependent. A discussion will be given below.

We note that a precise parameterization or simulations for higher temperatures are not accessible with the MC1 model due to the very long computation time. On a personal computer, a single simulation run for *T* = 300 °C using the above parameters takes about one week. This is caused by the large simulation fields in combination with the very high number of diffusion processes due to the small $E_{S}$. Simulations for a higher temperature with even larger simulation fields and a much higher *D* would require a substantially longer simulation time.

To speed-up the Monte Carlo simulation, in the modified simulation model MC2 we replace monomer diffusion via a large number of nearest-neighbour hops by fewer jumps over longer distances. This approximation is established by DeVita et al. as a multiscale kinetic Monte Carlo algorithm [32]. In detail, Equation (6) indicates that for $D\gg m_{n}F+\sum_{s}n_{s}R_{E,s}$ an individual monomer performs much more diffusion events compared to arrival plus escape. To reduce the number of simulation steps, all diffusion events within the time interval $\tau_{I}=1/\left(m_{n}F+\sum_{s}n_{s}R_{E,s}\right)$ up to the occurrence of the next arrival or escape are summarized. The monomer surface diffusion length is $\lambda =\sqrt{\tau D}$ according to the Einstein relation, with the diffusion time τ. In the time interval $\tau_{I}$ the diffusing monomer travels a distance $d_{I}=\sqrt{\tau_{I}D}$. Or, in other words, the rate $R_{I}=1/\tau_{I}=m_{n}F+\sum_{s}n_{s}R_{E,s}$ for travelling a distance $d_{I}$ by diffusion is given by the time interval up to the next arrival or escape. If there are other objects at a distance smaller than $d_{I}$, the diffusion can result in either a displacement of the monomer, in nucleation by collision with another monomer, or in attachment to a droplet. The probability *p* for a collision with another object depends on the circular segment r/(πd) covered by the object, where *d* is the distance and *r* is the radius of the object. This gives the nucleation rate $R_{N,ij}=rD/\left(\pi d_{ij}^{3}\right)$ at which monomer *i* collides with a second monomer *j*, with the distance $d_{ij}$ between both. Accordingly, the rate of attachment to a droplet *k* is $R_{A,ik}=rD/\left(\pi d_{ik}^{3}\right)$. Replacing the rate $n_{1}D$ for monomer diffusion in Equation (6) by the above modifications yields for the total activity rate:

(7) $$R_{tot}=m_{n}F+\sum_{s}n_{s}R_{E,s}+\sum_{i}\left(R_{I}+\sum_{j\neq i}R_{N,ij}+\sum_{k}R_{A,ik}\right)$$

As for the MC1 model, $R_{tot}$ is used for the random selection of the next process and the calculation of $dt=1/R_{tot}$.

With the modified MC2 model, simulation runs are performed for Al droplets on a 1400 × 1400 grid using the energy values $E_{S}$ = 0.19 eV and $E_{E}$ = 1.71 eV as for the rate model and MC1. In the temperature range of 150 °C ≤T≤ 300 °C the simulated Al droplet densities agree within ±4% to the results of the MC1 simulation. This indicates that the approximations applied for the multiscale KMC algorithm MC2 are compatible with the conventional MC1 model. As a huge advantage, a corresponding simulation run takes about 20 s, which is about 30,000 times faster than the MC1 simulation. This extreme improvement of the computation time suggests the modifications applied for MC2 also for processes at higher temperatures.

**Figure 6:**
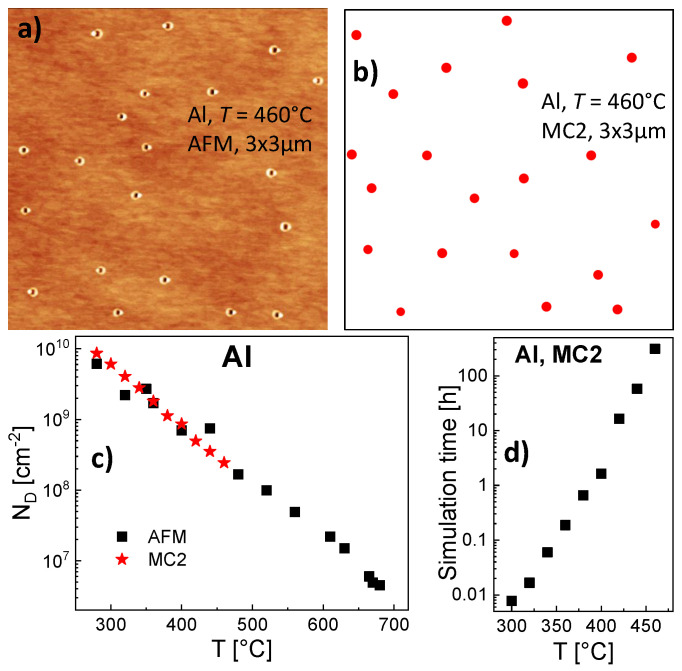
(**a**) 3 × 3 µm AFM image of an AlGaAs surface after Al droplet deposition at *T* = 460 °C. At this temperature, the deposited droplets are transformed into nanoholes during post-growth annealing (droplet etching). (**b**) Surface with Al droplets simulated using the MC2 model with $E_{S}$ = 0.19 eV, $E_{E}$ = 1.44 eV on a 7500 × 7500 field (3 × 3 µm). (**c**) Comparison of experimental Al droplet densities with simulation results obtained using MC2. (**d**) Time needed for a simulation run as function of the modeled process temperature.

A parameterization of the modified MC2 simulation for *T* = 300 °C using a 1400 × 1400 field yields agreement with the experimental droplet density for $E_{S}$ = 0.19 eV and $E_{E}$ = 1.44 eV (Figure 6c). Similar to the rate model (Figure 3a), the influence of $E_{S}$ is only weak. Further simulations using the same $E_{S}$ and $E_{E}$ demonstrate also very good agreement with the experimental droplet densities at higher *T* (Figure 6c). Due to the more than exponential increase of the time needed for simulation runs as function of *T* (Figure 6d), the model is limited for Al droplets to T≤ 460 °C. For illustration, Figure 6a shows an AFM image of an AlGaAs surface after Al droplet deposition at *T* = 460 °C. At this temperature, the deposited droplets are transformed into nanoholes during post-growth annealing [35]. Figure 6b shows a corresponding surface with droplets simulated with MC2.

As an interesting point, the value of $E_{E}$ = 1.44 eV determined with MC2 is much smaller than the $E_{E}$ = 1.71 eV given by the rate model. In order to examine the origin of the deviating energy parameters, we study the relevant processes in more detail. The droplet density is balanced by nucleation (collisions between mobile monomers) and dimer break break-off (monomer escape from dimers). To separate both processes, we have performed simulations for irreversible aggregation, where escape processes are frozen by a very high $E_{E}$ = 10 eV and droplet nucleation is controlled only by $E_{S}$. Here, for Al droplets, *T* = 460 °C, and $E_{S}$ = 0.19 eV, the rate model yields $N_{D}$ = 2.8 × 10${}^{10}$ cm${}^{-2}$ and the MC2 simulation a nearly identical $N_{D}$ = 2.6 × 10${}^{10}$ cm${}^{-2}$. The KMC model simulates nucleation basing on a more realistic atomistic picture, where diffusing monomers located on individual positions can collide. Here, the very small deviation between KMC and rate model results justifies the approximations made by the rate model, where the nucleation rate is calculated via $N_{1}^{2}\sigma_{1}D$ assuming an average monomer density and a capture number which describes the monomer depletion around other objects.

Now we switch to reversible aggregation with $E_{E}$ = 1.71 eV according to the rate model. Here the rate model yields $N_{D}$ = 2.4 × 10${}^{8}$ cm${}^{-2}$ in agreement with the AFM data, whereas the MC2 simulation computes a much higher $N_{D}$ = 9.2 × 10${}^{9}$ cm${}^{-2}$. This example demonstrates that the difference between rate model and MC2 simulation is related to dimer break-off. The rate model calculates dimer break-off via $2\pi r\zeta N_{2}D_{E}$ and, after escape, the monomers increases the average monomer density on the whole surface. For the atomistic Monte Carlo simulation, after escape, a monomer is still in the close proximity of the other monomer with a high probability for a re-nucleation and, thus, a recovery of the dimer. This effect causes an effective reduction of the rate of dimer break-off events for the KMC simulation and finally results in much higher droplet densities compared to the rate model. We note, that we consider the atomistic approach of the KMC simulation to be more realistic.

**Figure 7:**
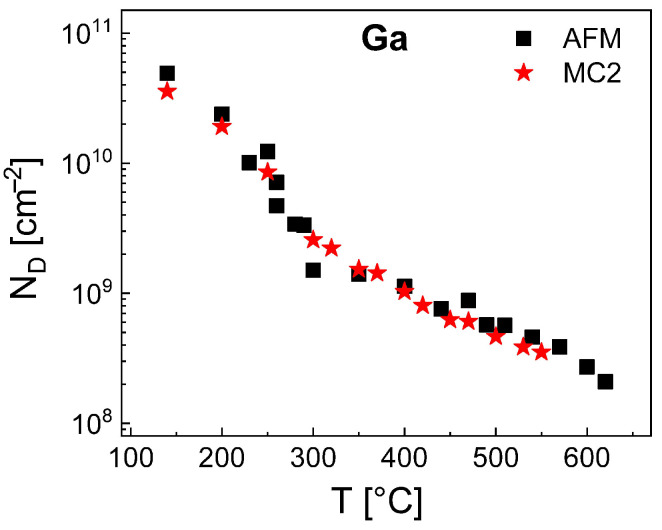
Comparison of experimental Ga droplet densities with simulation results obtained using the MC2 model with $E_{S}$ = 0.115 eV and a *T*-dependent $E_{E}$ (see text).

Finally, we have simulated the density of Ga droplets using the MC2 model. Since for Al droplets the value of $E_{S}$ is model-independent, we use here $E_{S}$ = 0.115 eV as given by the rate model. Furthermore, the results of the rate model establish that for Ga the value of $E_{E}$ depends on the temperature (Figure 4). A parameterization of the MC2 model yields for T≤ 300 °C a constant value of $E_{E}$ = 1.24 eV and for T> 300 °C a *T*-dependent $E_{E}$ = 1.24 + 0.06 (*T*[°C] − 300)/100 eV. These values of $E_{S}$ and $E_{E}$ allow a very good reproduction of the Ga droplet density by MC2 as is demonstrated in Figure 7. Like for the MC2 simulations of the Al droplet density, the values of $E_{E}$ are smaller than those of the mean-field model ($E_{E}$ = 1.51 eV for T≤ 300 °C and $E_{E}$ = 1.51 + 0.1 (*T*[°C] − 300)/100 eV for T> 300 °C). We assume that the above re-nucleation effect also reduces the effective rate of escape processes from Ga droplets and requires a lower $E_{E}$ in comparison to the mean-field model. The accessible temperatures up to 550 °C are higher compared to MC2 simulations of Al droplets which is caused by the lower densities of Ga droplets and the, thus, reduced simulation fields.

## 7. Conclusions

The deposition temperature controls the density of Ga and Al droplets during droplet epitaxy and etching over several orders of magnitude. This allows the self-assembled creation of quantum emitters with a tailored feature density. In particular ultra-low densities around 10${}^{7}$ cm${}^{-2}$ are interesting for applications in quantum information technology, where individual quantum emitters can be easily addressed. The droplet density is balanced by nucleation events caused by collisions between diffusing adatoms and the escape of atoms from droplets. Both processes are characterized by the material dependent activation energies $E_{S}$ for surface diffusion and $E_{E}$ for escape.

The present manuscript studies the applicability of three different theoretical approaches for the modeling of the temperature-dependent density of Ga and Al droplets. An analysis basing on a scaling law requires the knowledge of the critical nucleus size *i*. Since for the droplets studied here, the value of *i* depends on temperature and varies with the deposition time, a simple scaling analysis is questionable. A model using mean-field rate equations is able to quantitatively reproduce the experimental Al droplet densities over the whole temperature range using $E_{S}$ = 0.19 eV and $E_{E}$ = 1.71 eV. This is an important result and demonstrates that the temperature dependent nucleation is balanced by the kinetics of droplet nucleation and the escape of atoms from droplets. Other processes such as Ostwald ripening as assumed earlier [12] are not relevant. Furthermore, the small value of $E_{S}$ indicates a very high activity of the mobile Al adatoms on the surface. In contrast to that, for Ga droplets the experimental droplet densities can be reproduced by $E_{S}$ = 0.115 eV, $E_{E}$ = 1.51 eV only at low temperatures up to 300 °C, higher temperatures require a *T*-dependent $E_{E}$. This result establishes that for T> 300 °C additional processes become relevant that modify the binding energy of adatoms to the droplets.

In a further approach for modeling, the Al droplet density is studied using a kinetic Monte Carlo (KMC) simulation basing on an atomistic representation of the mobile adatoms on the surface. A conventional KMC model requires very long computation times for modeling high process temperatures with high surface activity and low droplet density. Using a multiscale KMC algorithm [32] to shorten the computation times, the KMC simulation quantitatively reproduces the Al droplet densities up to *T* = 460 °C. Interestingly, the value of $E_{S}$ = 0.19 eV is equal to the result obtained using the rate model, but the value of $E_{E}$ = 1.44 eV is much smaller. This is attributed to the mean-field assumption of the rate model, where adatoms after escape from a droplet increase the average droplet density over the whole surface. In contrast to that, in the KMC model the escaped atoms are still in the close proximity of the droplet with a high probability of a recapturing. This reduces the effective rate of escape processes and, thus, requires a lower $E_{E}$ in comparison to the mean-field model.

We conclude that a KMC model is more accurate for modeling of droplet nucleation compared to a mean-field rate model. On the other side, the computation times of the rate model are substantially shorter which allows the modeling also of high process temperatures. Therefore, a modified rate model which considers the spatial distribution of adatoms after escape from a droplet is highly desirable.

## Data Availability

Not applicable.
